# Body mass index changes: an assessment of the effects of age and gender using the e-norms method 

**DOI:** 10.1186/s12874-021-01222-z

**Published:** 2021-02-22

**Authors:** Joe F. Jabre, Jeremy D.P. Bland

**Affiliations:** 1 Department of Neurology, David Geffen School of Medicine at UCLA, Los Angeles, CA USA; 2 Dept of Clinical Neurophysiology, East Kent Hospitals University NHS Foundation Trust, Canterbury, UK

**Keywords:** Body Mass Index, Normal Values, E-norms, Obesity, Age Sex

## Abstract

**Background:**

To validate e-norms methodology in establishing a reference range for body mass index measures. A new method, the extrapolated norms (e-norms) method of determining normal ranges for biological variables is easy to use and recently was validated for several biological measurements. We aimed to determine whether this new method provides BMI results in agreement with established traditionally collected BMI values.

**Methods:**

We applied the e-norms method to BMI data from 34,384 individuals and compared the ranges derived from this method with those from a large actuarially based study and explored differences in the normal range by gender, and age.

**Results:**

The e-norms derived range of healthy BMI in adults is from 22.7 to 30.6, and showed that BMI is consistently higher in men than in women and increases with age, except in subjects aged 80–98 years in whom healthy BMI appears to be lower.

**Conclusions:**

Our e-norms derived healthy BMI ranges agree with traditionally obtained actuarially based methods, supporting the validity and ease of use of our method.

## Background

The US CDC, [1] in common with the WHO, [2] defines a BMI range from 18.5 to 24.9 as “Normal or healthy weight” for adults over 20 years of age, and a BMI > 30 as ‘obese’.

These threshold values are derived from historical studies of actuarial insurance databases and more recent studies of large epidemiological databases collected in comprehensive healthcare systems. These demonstrate that BMI values outside this range are associated with an increased incidence of either all causes mortality, [3, 4] or specific conditions such as Hodgkin’s lymphoma.

In this work, we propose to use a novel technique, the extrapolated norms or e-norms method to calculate BMI normative values from BMI data that contains both normal and abnormal values collected in our practice.

The method relies on a behavior we refer to as “e-norms clustering” [5] that reveals a narrow range between the minimum and maximum values of a variable obtained from normal individuals as compared to a variable obtained from individuals with pathology that displays a much larger minimum to maximum range. This e-norms clustering behavior is independent of the type of pathology being investigated and will be described in more detail in the e-norms method section below.

To date, the e-norms method has been validated by various authors for neurophysiological and non-neurophysiological data. This work represents our first attempt to use it in evaluating Body Mass Index (BMI) normative data.

This work was undertaken to show that normative data extracted from a mixed dataset using the e-norms method yields similar results to existing traditionally obtained BMI estimates derived from actuarial data that are costly to conduct and require long-term follow-up of many subjects.

Despite long-established WHO recommendations on healthy BMI, there remain uncertainties regarding the interpretation of BMI. A recent study of 3.6 million individuals concluded - “…further work is needed to establish whether increased weight is actually beneficial for older individuals.” [3] There is also evidence that a single range for healthy BMI is not appropriate for all ethnic groups, [6, 7] but large, long term prospective studies are especially difficult to conduct in these subpopulations. Even the largest recent studies leave residual uncertainty in their conclusions as to whether healthy BMI has the same range in different age groups, notably the elderly [8]. These shortfalls notwithstanding, the age groups we chose to calculate BMI normal values for were selected for comparing our results to those derived from an actuarial study of 3.6 million individuals in the UK [3].

## Methods

We applied the e-norms method to BMI data derived from 34,384 individuals we collected in our practice between 1994 and 2019 to compare the ranges we derive by this method to those obtained from a large actuarially based study of 3.6 million individuals identifying the incident disease, and the ascertainment of death.

The source data for our study are the records of patients attending the clinical neurophysiology department in Canterbury, UK for investigation of possible carpal tunnel syndrome (CTS). Presence of significant risk factors and chronic conditions known to be risk factors for CTS were also collected. These included age, gender, height and weight, as well as occupational status and the presence or absence of thyroid disease, diabetes, acromegaly, arthritis and wrist trauma, smoking status, and family history of CTS.

The records include BMI because of a previously well documented relationship between high BMI and an increased incidence of CTS [9]. Indeed 57 % of the patients in our dataset were positive for CTS and 43 % were negative. The advantage our material presented was that it was collected by the same investigator (JDPB), in the same hospital, from the same patient referral pool, using the same collection and analysis methods.

We have generic ethics permission to use anonymized data from this database for our research. The research ethics approval was obtained from South Central (Hampshire A) National Research Ethics Service committee in the UK.

We extracted records made at the patient’s first presentation to the department for diagnosis, thus excluding follow-up visits and including each subject only once. We excluded subjects under 17 years of age and 65 subjects with missing data for height or weight. The extracted data fields for analysis were BMI, age, sex, and the presence or absence of laboratory confirmed CTS, the last being included only so that we would be able to conduct exploratory analyses of whether the disease specific nature of the population might influence the results. Thirty-four thousand and three hundred and eighty-four (34,384) BMI measures of 22,661 females and 11,723 males aged 17–98 years old were analyzed. We derived e-norms based healthy BMI estimates for the entire cohort; for males and females separately; and for four age groups in each (17–49, 50–69, 70–79 and 80–98 years). We chose these age groups to match those used in the study we were using for comparison [3]. Paired two sample t-tests were used to compare e-norms BMI values between males and females of the same age groups, both of which were Gaussian distributed.

### The e-norms method

The e-norms method [10] allows the use of data derived from a provider’s own cohort to produce normative values for any parameter in their database. The method has been validated by various authors for neurophysiological and non-neurophysiological studies ranging from electrodiagnostic studies, [11–15] to acetyl choline receptor antibodies (AchRAb) for diagnosing myasthenia gravis (Guan Y, unpublished data), and more recently to Ophthalmology, for deriving biometric normative data used for intraocular lens (IOL) power calculation prior to cataract surgery [16]. To date, normative data derived using the e-norms method in all these studies was found to closely match data obtained from traditional studies, producing much needed normal values in populations and cohorts for which none were available. The method has been proven particularly useful in pediatric cohorts where normal values change rapidly with age and works as follows:

A variable’s data is sorted in ascending order in an Excel spreadsheet and plotted against its rank order producing a cumulative distribution plot that reveals an inverted S curve consisting of a steep lower left; a flat or “plateau” middle; and a steep upper right.

First-order derivatives are then calculated for each successive data point by subtracting the second value from the first, the third from the second, and so on until all the differences between successive values have been calculated. The first-order derivatives are then plotted on the same graph as the sorted variables to help in identifying the plateau part of the curve, the one corresponding to the lowest first order differences, consistent with the e-norms clustering behavior.

The e-norms clustering behavior is data neutral and can be illustrated using blood glucose levels as follows:

Fasting blood glucose levels have a minimum to maximum range between 70 and 99 mg/dl, with a min to max difference a mere 29 mg/dl.

In diabetic patients, such differences are hundreds of times that range. The Guinness World Records lists “Michael Patrick Buonocore (as having) survived a blood sugar level of 2,656 mg/dl when admitted to the Pocono Emergency Room in East Stroudsburg, Pennsylvania on 23 March 2008” [17]. Since an abnormal fasting Blood Glucose can be as low 100 mg/dl, the min to max difference in patients with known diabetes can in theory be an astounding 2556 mg/dl, a much greater difference than in individuals with normal Blood Glucose. E-norms clustering leverages this behavior and can be used in identifying datasets derived from normal subjects from those derived from subjects with pathology.

**Fig. 1:**
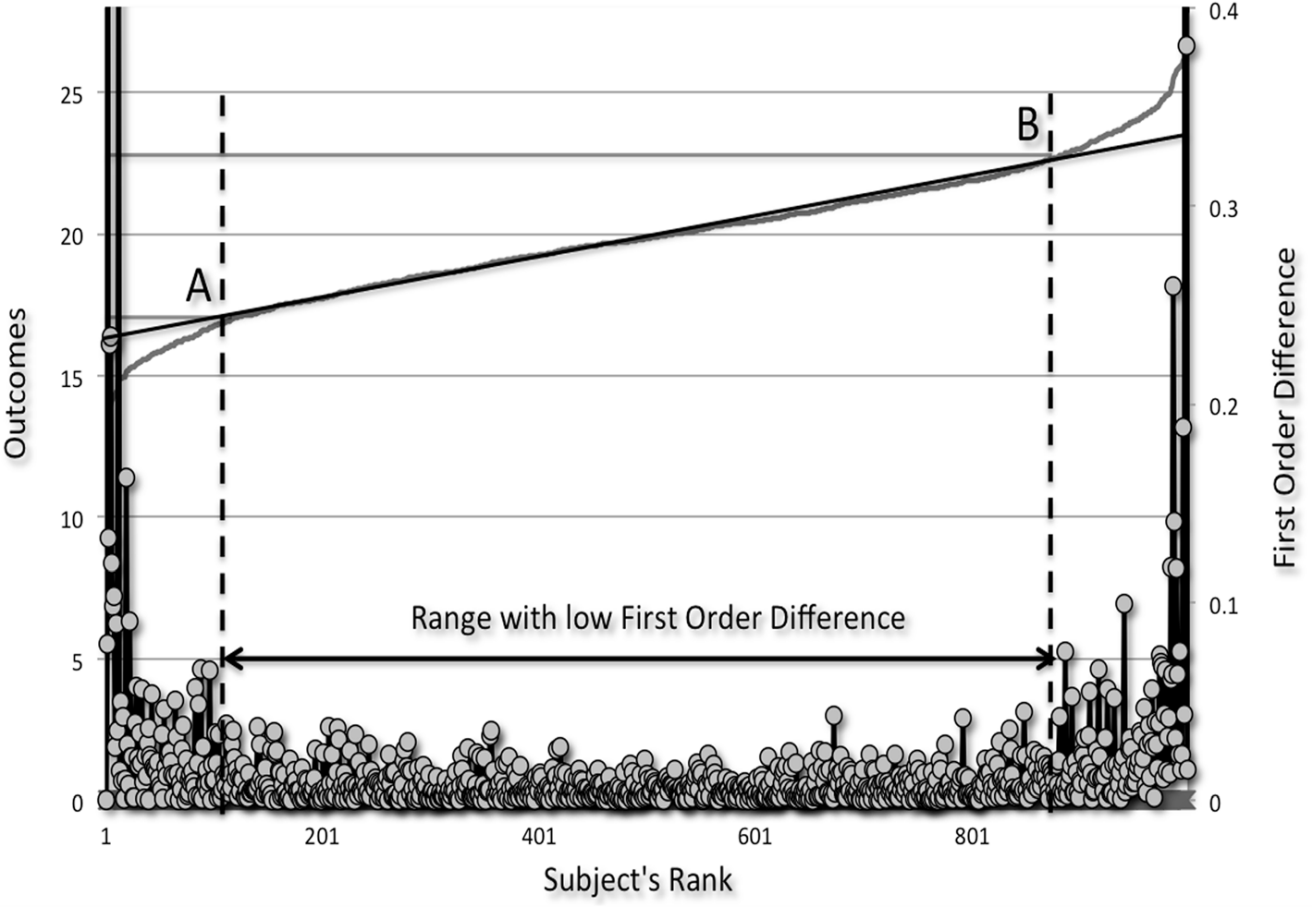
Sample e-norms plot. Cumulative density (curve) first-order difference (dots) of a simulated variable with 1,000 data points and a mean of 20 and standard deviation of 1.5. When a straight-line fit is overlaid on the inverted S curve, the plot of the first-order difference reveals the range of data points with low first-order difference, helping to identify inflection points A and B. Reprinted by permission from Wolters Kluwer Health, the Journal of Clinical Neurophysiology: Jabre JF, Pitt MC, Deeb J, Chui KKH. E-norms: a method to extrapolate reference values from a laboratory population. 2015;32(3):265–270. Copyright 2015 by the American Clinical Neurophysiology Society.

To illustrate this concept, we will use a simulation study that displays 1,000 simulated values that have a mean of 20 and a SD of 1.5, with a mean ± 4 SD value of 14 and 26, respectively. We will determine if we can extract the mean ± 2 SD values of 17 and 23 from this graph from the data that lies within the plateau part of the curve. The plot of this simulated data can be seen in Fig. 1.

Data points at the left and right extremes of the curve, display higher first order differences between them than those between points A and B that mark the curve’s points of inflection. Descriptive statistics of the data lying between points A and B within the plateau part of the curve reveals values that lie between 17 and 23 identical to the normal limits of the data as predicted by the mean ± 2 SD we set out to represent.

In a recently completed study, plateau identification and determination of the left and right inflection points of the e-norms plot has been proven reliable. Twenty different observers recruited from a diverse pool of hospital workers were asked to visually identify the e-norms plateau in 393 upper and 284 lower limb nerve conduction studies while blinded to the variable they were analyzing. An inter-rater ANOVA without replication testing showed no significant difference between their findings. [18].

A significant advantage of the e-norms method is that it can be performed in minutes using a Microsoft Excel spreadsheet that is uploaded anonymously and securely to an encrypted e-norms web application developed by one of the authors (JFJ) for this purpose [19].

## Results

**Fig. 2:**
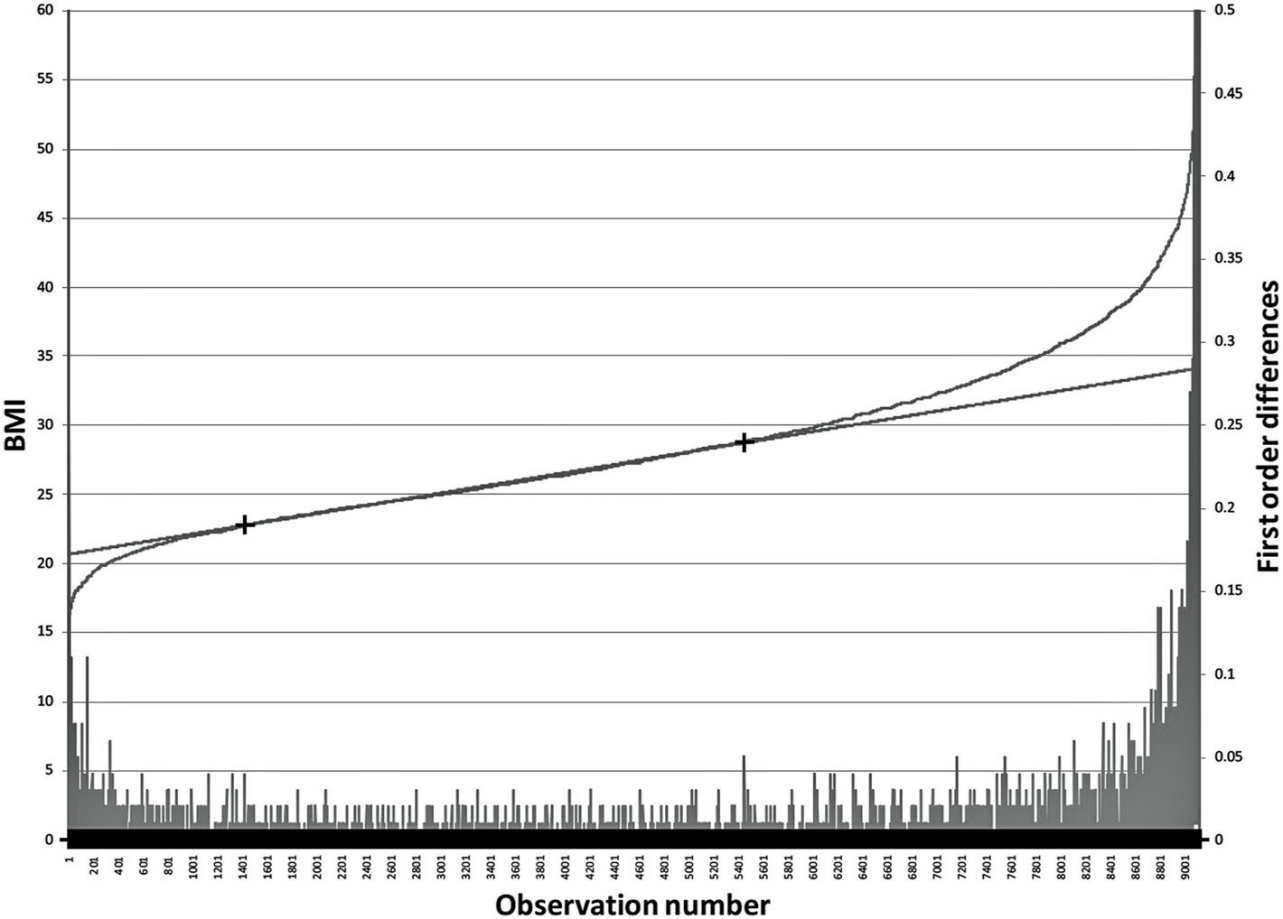
E-norms plot of BMI values. Cumulative density curve of 50-69 years old women BMI values. Points A and B on the cumulative density curve delineate the boundaries of the e-norms plateau. Note the plateau correspondance with the lowest first order differences.

The age group and sex distribution of the entire patient cohort is shown in Table 1 and the e-norms plot from our data is shown in Fig. 2.

**Table 1:** Demographics of the BMI source dataset

Age group	Males n	Females n
**17–49**	4053	8777
**50–69**	5016	9129
**70–79**	1726	2776
**80–98**	928	1979
**TOTAL**	**11,723**	**22,661**

The estimate of healthy BMI derived with the e-norms method for our entire dataset showed a mean of 26.5, and a standard deviation of 2.2 with a range from 22.7 to 30.6. The distribution is not normal (Skewness 0.07, Kurtosis − 1.09, Kolmogorov-Smirnov test d = 0.057 *p* < .01, Lilliefors *p* < .01).

**Fig. 3:**
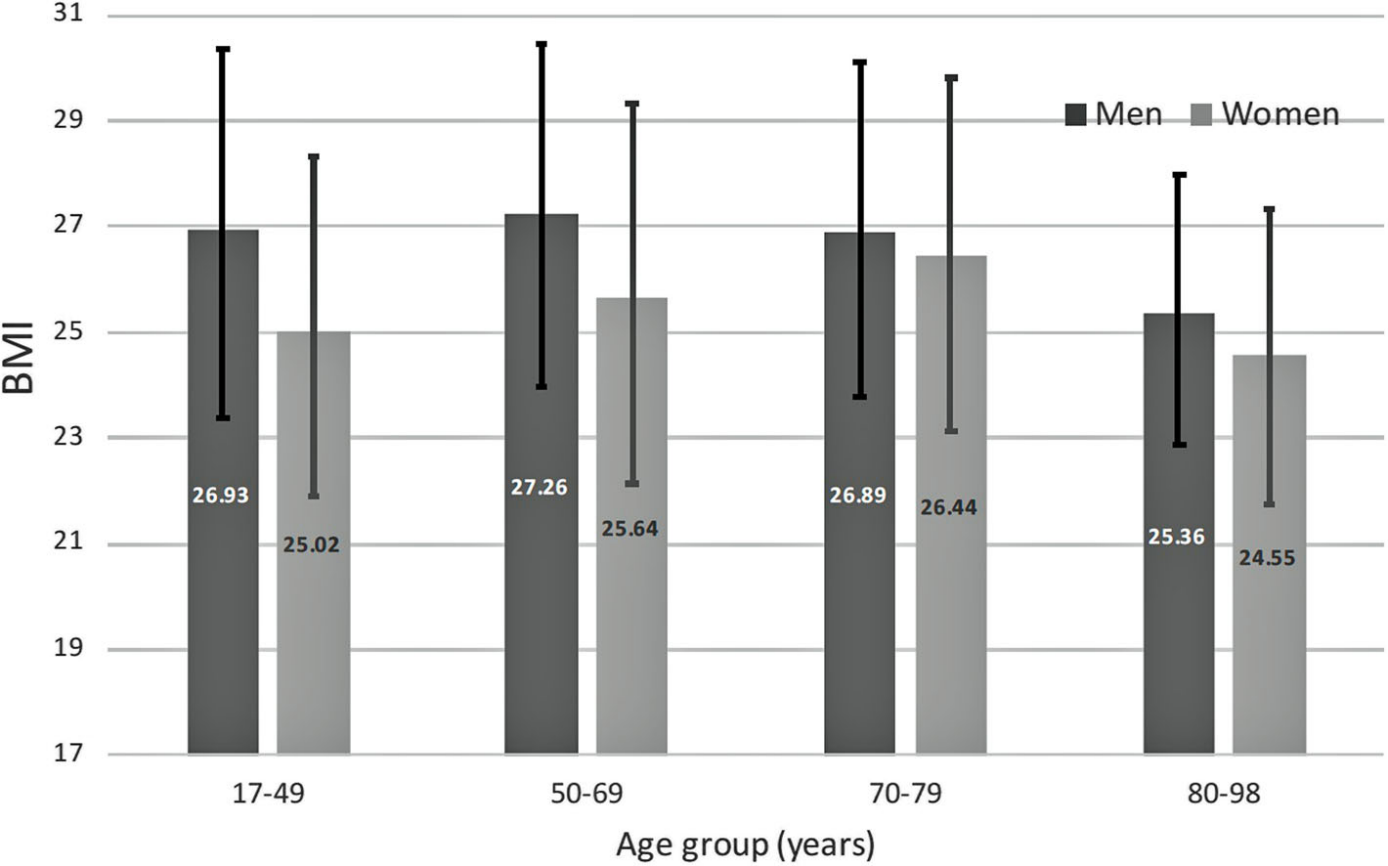
E-norms derived BMI normal values. Mean (bars) and minimum to maximum range (whiskers) of normal BMI values by sex and age derived by the e-norms method

E-norms derived estimates of healthy BMI in subgroups of the population displayed variations with age and sex as seen in Fig. 3.

In men it reaches a peak in the 50–69 age group but reveals a marked decrease in healthy BMI in the oldest age group. In women however, it reaches a peak in the 70–79 age group before showing the same marked decrease in the oldest members of the population. The ranges for men were consistently higher than those for women in all age groups, with the minimum and maximum differences being highest in the 17–49 and 50–69 age groups, and lowest in the 70–79 age group. All of the men to women differences were statistically significant at the *p* < .05 level using a paired t-test two sample analysis for means.

## Discussion

A simple web search for BMI normal values in sites ranging from the World Health Organization (WHO) [2], to the Center for Disease Control and Prevention (CDC) [1, 20] the American Cancer Society [21], the American Heart Association [22], and the NHS [23], lists BMI normal values as indicative of underweight, for BMI less than 18.5; normal weight; for BMI between 18.5 and 24.9; overweight, for BMI between 25 and 29.9; and obese, for a BMI of 30 or more.

Numerous studies have investigated the relationship between obesity and life expectancy by studying the relationship between BMI measures and mortality. But the evidence of this remains inconclusive varying from none [24], to an inverse relation [25], or a direct one [26] due to differences in analyses methods using U versus J shaped relations. Wong et al [27] used a method they refer to as multivariable fractional polynomials (MFP) to “determine the best fitting functional form for BMI .. to capture the relationship between mortality and BMI in a compact, parsimonious model.”

Given these inconsistencies, an easy breakdown of BMI normal values by age and gender that does not require long and costly actuarial studies will add great value to the investigation of these relationships. Our work set out to investigate the use of this novel approach to derive normative BMI measures broken down by age and gender, and aimed to determine whether this method provides BMI results in agreement with established and traditionally collected BMI values. Our 30.6 BMI estimate of the upper limit of healthy BMI derived using the e-norms is remarkably close to the currently accepted threshold of obesity. This value also corresponds quite closely to the level at which the hazard ratio for all-causes mortality in the UK population of 3.6 million individuals rises above 1.0 with increasing BMI.

Where underweight is concerned however, the e-norms estimate of the lower boundary of healthy BMI of 22.7 is markedly higher than the WHO suggested figure of 18.5. When examining the data for all-causes mortality in Bhaskaran et al’s Fig. 1 however, it is clear that there is a sharp increase in mortality as BMI falls, long before the WHO threshold of 18.5 is reached. The hazard ratio for all-causes mortality in that study rises above 1 at a BMI of approximately 22. To that end, unlike with traditional methods, the e-norms analysis has estimated a lower boundary for healthy BMI that actually corresponds to the level at which all causes mortality becomes significantly elevated.

This leads us to believe that the current WHO definition of ‘underweight’ as BMI < 18.5 is too conservative and that the adverse health implications of low BMI have perhaps received insufficient attention.

Having established that our estimate of the range of healthy BMI corresponds closely to the range of lowest all-causes mortality we then set out to examine if our e-norms estimates support earlier suggestions regarding variation in healthy BMI by age and sex.

Data from Bhaskaran et al’s Fig. 3 suggests that the range of healthy BMI may be wider with an extended upper limit in women compared to men, although this data was limited to the subset of ‘never smokers’. [3] In smokers however, several workers [28–30] have shown a higher risk for pregnant smokers with obesity and adverse maternal and infants birth outcomes.

Earlier studies have suggested that the BMI associated with minimal mortality rises gradually with age, from about 22 at age < 50 to 25 at age > 80 years. [3] A systematic review concluded that the optimal BMI range for the elderly was between 25 and 35 and that the relationship between BMI and mortality is weaker in older age groups. [8].

Our finding that ‘normal’ BMI in the very elderly is noticeably lower than the figure suggested by actuarial studies could be accounted for by a high prevalence of chronic pathologies in this age group that, either by direct effects (diabetes) or indirect ones (relative inactivity as a result of arthritis for example), are associated with higher BMI, thus introducing an overall upward bias into BMI measures in this age group when they are studied by conventional actuarial methods.

One cannot exclude however that survivor bias may account for our finding in the > 80 years age group. In this scenario, individuals who reached this advanced age were probably healthier, and likely had a lower BMI earlier than those who didn’t. The e-norms method would therefore not be determining the healthy BMI range for a hypothetical aged 80 + population but for a healthy sub-population of survivors in this age group. It would be interesting to study mortality data in relation to BMI in demonstrably healthy elderly individuals who have been screened for the absence of common chronic disease.

Such a study would be handicapped however by the intrinsic difficulty of following up patients in the their 80 s and above for the long periods required for mortality studies. In the meantime, we would suggest that it is not safe to assume that a BMI of 31 in an 80-year-old is necessarily of less concern than a similar measurement in a 30-year-old.

## Conclusions

We investigated whether the e-norms method can be applied to develop BMI, considered here as just another biological marker. The e-norms method is a recently developed technique that has been successfully applied in other disciplines to derive normative data from a laboratory population. The close agreement between our e-norms estimates of the upper limit of a normal BMI and those derived from the literature at which all-causes mortality begins to rise markedly, serves as validation of this technique. The strengths of our study include a relatively large source dataset and an entirely different analytical approach to existing actuarial studies of healthy BMI that are costly to perform and require long-term follow-ups. Although the source population in our cohort were all suspected of having CTS, we are mindful that by no means they represent a random sample of the population. But given the close correspondence between our estimates of healthy BMI and those derived from mortality studies, we believe that our assumptions are valid and would allow practitioners with limited time and resources to take advantage of existing datasets to determine the range of normal BMI in their own cohorts in a fast and efficient manner.

## Data Availability

The datasets used and/or analyzed during the current study available from the corresponding author on reasonable request.

## Abbreviations

E-norms: Extrapolated norms
BMI: Body Mass Index
US CDC: United States Center for Disease Control and Prevention
WHO: The World Health Organization.
CTS: Carpal tunnel syndrome
AchRAb: Acetyl choline receptor antibodies
IOL: Intra-ocular lens
SD: Standard Deviation
